# Localization of β-Nerve Growth Factor in the Stallion Reproductive Tract

**DOI:** 10.3390/vetsci11080367

**Published:** 2024-08-12

**Authors:** Alison Mickelson, Forgivemore Magunda, James Graham, Patrick McCue

**Affiliations:** 1 Department of Biomedical Sciences, Colorado State University, Fort Collins, CO 80521, USA; 2 Department of Microbiology, Immunology and Pathology, Colorado State University, Fort Collins, CO 80521, USA; 3 Department of Clinical Sciences, Colorado State University, Fort Collins, CO 80521, USA

**Keywords:** nerve growth factor-β, stallion, reproductive tract, immunohistochemistry

## Abstract

**Simple Summary:**

The goal of this research was to determine if the neurotrophin β-Nerve growth factor is present in the reproductive tract of stallions. This protein has been identified in the semen of camelids and functions to stimulate the release of a hormone from the pituitary of female camelids that is ultimately responsible for the induction of ovulation.

**Abstract:**

β-Nerve growth factor (β-NGF) is a protein produced in the reproductive tract of camelids (camels, llamas, and alpacas) that has been identified as the ovulation inducing factor in seminal plasma. β-NGF from seminal plasma deposited into the reproductive tract of the female camelid acts systemically to stimulate the secretion of luteinizing hormone (LH) from the anterior pituitary, which in turn induces follicle maturation and ovulation. The objectives of the present study were to determine if β-NGF is present in the reproductive tract of the stallion and identify the specific site(s) of production. The hypotheses were that β-NGF would be present in the stallion reproductive tract and would primarily be localized in Sertoli cells of the testes and the prostate gland. Immunohistochemistry on paraffin-embedded paraformaldehyde-fixed tissues was performed using a rabbit polyclonal anti-β-NGF antibody on a total of six male equine reproductive tracts, including a one-day old colt, a one-year-old colt, and four adult stallion tracts. Strong immunostaining was observed in the efferent ducts of the testes and the epithelial cells of the prostate, seminal vesicles, bulbourethral glands, and ampullae. Weaker β-NGF staining was noted in Leydig cells, Sertoli cells, and spermatogonia within the testes and in epithelial cells of the epididymis. In conclusion, immunohistochemistry revealed that β-NGF is present in the stallion reproductive tract, and the protein is primarily present in the efferent ducts of the testes and in all accessory sex glands.

## 1. Introduction

Seminal plasma is largely secreted from the accessory glands with a lesser portion contributed by the epididymides and testes. The stallion accessory glands include paired ampulla surrounding the vas deferens, paired seminal vesicles, prostate gland, and a bulbourethral gland [1,2]. Bulls and stallions have all four accessory sex glands, whereas camelids do not have seminal vesicles [1,3]. Accessory sex glands differ between species by size and secretions, as well as anatomical and histological structure [1]. Seminal plasma has been reported to affect spermatozoa transport, motility, and capacitation, as well as cause stimulation of uterine contractions in the female [1,3,4]. In addition, the seminal plasma of male camelids have been reported to contain an ovulation-inducing factor (OIF) that stimulates female camelids to ovulate after mating [5,6,7].

The ovulation-inducing factor was first observed in Bactrian camels [5]. Administration of seminal plasma by intravaginal, intramuscular, or intrauterine routes caused ovulation in female camels [5,6,7]. Similarly, intramuscular administration of seminal plasma to female llamas and alpacas resulted in an ovulation rate of more than 90% [8]. The ovulation rate observed was similar to that of female llamas after administration of gonadotropin-releasing hormone (GnRH) or following natural service. The physiologic mechanism by which OIF triggers ovulation is the stimulation of a surge of luteinizing hormone (LH) from the anterior pituitary [8]. Camelid seminal plasma was also noted to have a luteotrophic effect as plasma progesterone concentrations were higher, the corpus luteum (CL) tended to grow for a longer period, and the diameter of the CL was greater in llamas administered seminal plasma [8].

The ovulation-inducing factor has been determined to be structurally identical to the protein β-Nerve Growth Factor (β-NGF) [9]. β-NGF belongs to a family of neurotrophins which typically exist in nature as a homodimer with a molecular mass of 26–27 kDa [3,9]. The nerve growth factor has an essential role in promoting survival, maintenance, and growth of sensory (dorsal root) and sympathetic neurons and cells of the adrenal medulla [3,10].

NGF was originally identified in mouse sarcoma, cobra venom, and adult male mouse submaxillary glands [3,10,11], but was subsequently identified in reproductive tissue, including the prostate gland of guinea pigs [12]. Further studies have reported that NGF was localized in at least one male accessory sex gland, with the primary source being the prostate in llamas, the vesicular gland and ampullae in bovids (cattle and bison), and the ampullae and prostate in cervids (elk and white-tailed deer) [12].

Limited research has been performed on the presence or function of β-NGF in horses. Bogle and coworkers injected female llamas with equine seminal plasma, which resulted in ovulation of 22 to 38% of treated animals depending on dose administered [13]. Druart and colleagues identified β-NGF in seminal plasma from stallions using two-dimensional liquid chromatography-tandem mass spectrometry (2DLC MS/MS) [4].

The objective was to present preliminary results of a study to determine if β-NGF protein is present in the reproductive tract of the stallion and to identify the specific site(s) of production. The hypotheses were that β-NGF would be present in the stallion reproductive tract and that production would primarily be localized in the Sertoli cells of the testis and in the prostate gland.

## 2. Materials and Methods

### 2.1. Tissue Preparation

Reproductive tracts were harvested from 6 male horses that were euthanized for unrelated medical conditions at the Veterinary Teaching Hospital, Colorado State University. Tissues were acquired from four adult stallions, one colt approximately one year of age and one colt foal that was one day old. Reproductive tissues were collected during necropsy and included testes, head, body, and tail of the epididymis, vas deferens, ampullae, seminal vesicles, prostate, and bulbourethral gland. Alpaca testes and equine cornea were collected as positive control tissues. All tissues were fixed for at least 24 h in 4% paraformaldehyde in phosphate-buffered saline (PBS) and subsequently embedded in paraffin blocks. Tissues were then processed to obtain 5 μm paraffin sections on glass slides. Additional non-reproductive tract tissues harvested and processed included the heart, lung, skeletal muscle, kidney, and spleen from an adult stallion.

### 2.2. Immunostaining

Slides containing tissue sections were washed three times in xylene and twice in absolute ethanol. Slides were then submerged in a successive gradient of ethanol solutions (90%, 70%, 50%, and 30%) followed by two washes with deionized water, and then were submerged in citrate buffer solution (pH 6.0) and incubated in a pressure cooker for 3 min. Slides were subsequently cooled to room temperature for 30 min and washed twice in deionized water. A general block (5% normal goat serum in universal blocking solution made from bovine serum albumen) was applied to the tissue for 60 min, followed by treatment with hydrogen peroxide blocking solution (0.05% hydrogen peroxide) for 15 min. The primary antibody used for immunohistochemical localization of NGF was polyclonal rabbit antiserum raised against human NGF (PA5-14872; Thermo Fisher Scientific, Waltham, MA, USA). The antibody was diluted 1:100 in antibody diluent (64211; Abcam, Cambridge, UK). Slides were incubated overnight at 4 °C in a humidified chamber and then washed three times for 5 min with wash buffer solution (1% TBS and 0.005% Triton™ X in deionized water). Tissues were then incubated in a humidified chamber with the secondary antibody (goat anti-rabbit IgG-HRP; G-21234; Thermo Fisher Scientific, Waltham, MA, USA) diluted 1:500 in antibody diluent (64211; Abcam, Cambridge, UK). A 3,3′ diaminobenzidine (DAB) substrate kit (64238; Abcam, Cambridge, UK) was used for color development, and hematoxylin was used as a counter stain. Slides incubated with antibody diluent, but without the primary antibody, served as negative controls. An β-NGF protein block was applied to all reproductive tissue slides with the primary antibody to test the specificity of the antibody. Slides containing equine cornea and alpaca testes were used as positive controls. All test tissues and controls were stained simultaneously for controlled timeframes to ensure the uniformity of staining. Histology was performed on serial sections stained with hematoxylin and eosin to evaluate morphology.

## 3. Results

**Figure 1:**
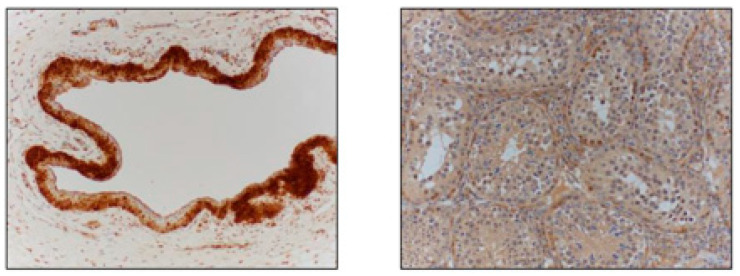
Staining of positive control tissues, including equine cornea (**left image**) and alpaca testis (**right image**).

**Figure 2:**
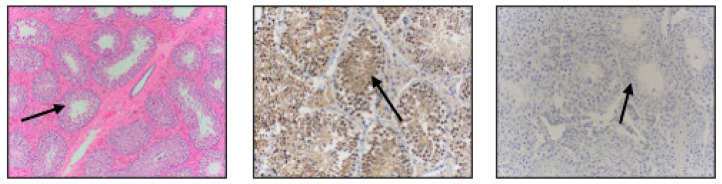
Stallion testicular tissue stained with hematoxylin and eosin (**left image**), immunostained for β-NGF (**middle image**), and negative control (**right image**). Arrows point to the seminiferous tubules.

**Figure 3:**
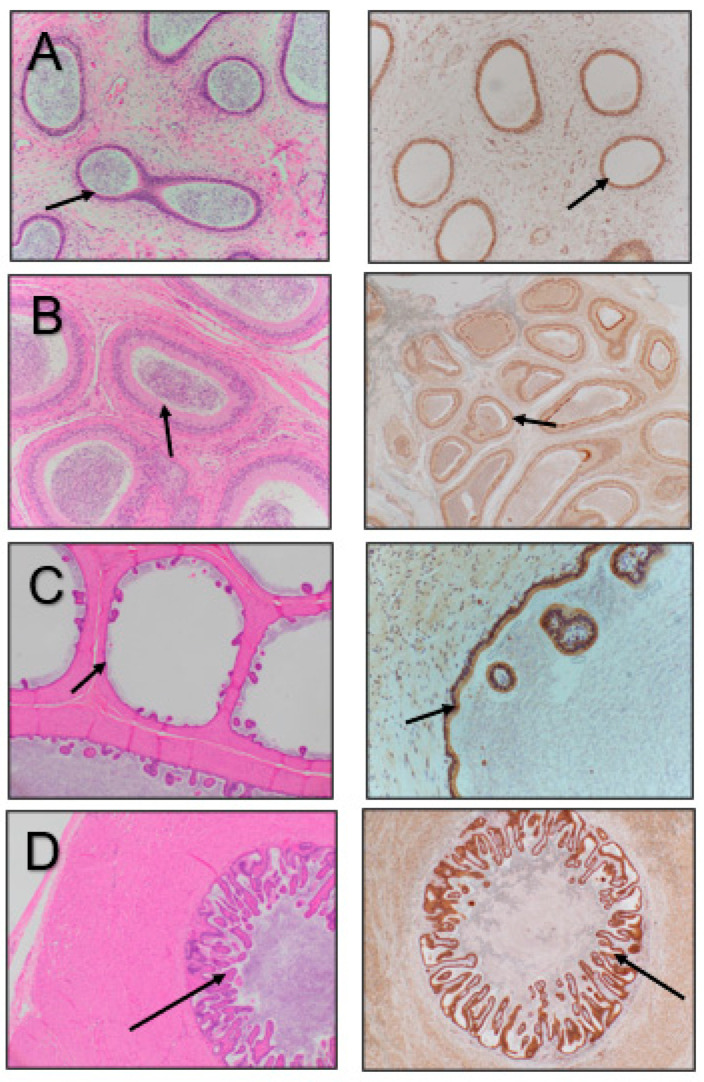
Epididymal tissue stained with hematoxylin and eosin (**left images**) and immunostained for β-NGF (**right images**). (**A**) Head of epididymis, (**B**) body of epididymis, (**C**) tail of epididymis, (**D**) vas deferens. Arrows point to the luminal epithelium of the various structures.

β-NGF was identified in both positive control tissues (equine cornea and alpaca testis; Figure 1) and tissues from all 6 male equine reproductive tracts. Negative controls reacted appropriately. Tissues from all reproductive organs exhibited positive immunostaining, but staining intensity for β-NGF differed between tissues (Table 1). In the testes, the strongest staining was in the efferent ducts and lighter staining was observed in interstitial (Leydig) cells, Sertoli cells and spermatogonia (Figure 2). In the interstitial (Leydig) cells, a majority of immunostaining was located in the apical region, with more limited immunostaining in the nucleus and cytoplasm. The head, body, and tail of the epididymis, as well as the vas deferens, exhibited apical immunostaining of the epithelial cells (Figure 3). Intensity of staining increased distally with the strongest immunostaining observed in the tail of the epididymis and vas deferens. Tails of spermatozoa in the body region of the epididymis also appeared to stain positive for β-NGF.

**Figure 4:**
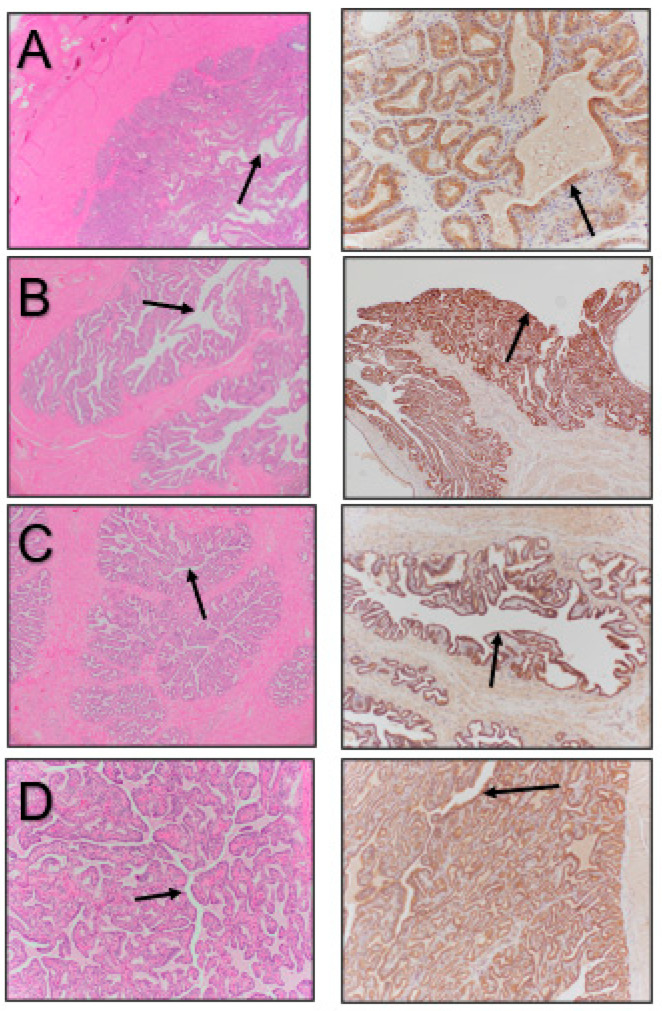
Accessory sex glands stained with hematoxylin and eosin (**left images**) and immunostained for β-NGF (**right images**). (**A**) Ampulla, (**B**) seminal vesicle, (**C**) prostate, (**D**) bulbourethral gland. Arrows point to the luminal epithelium of the various structures.

Accessory sex gland tissue exhibited more staining than tissues from the other reproductive organs, aside from the efferent ducts of the testes. The ampullae, seminal vesicles, prostate, and bulbourethral glands all exhibited strong apical staining (Figure 4). The disseminate and body of the prostate gland stained with the same intensity. The strongest apical staining was observed in the prostate and seminal vesicles. The prostate and bulbourethral glands also exhibited cytoplasmic staining along with limited staining of the nucleus and smooth muscle.

**Figure 5:**
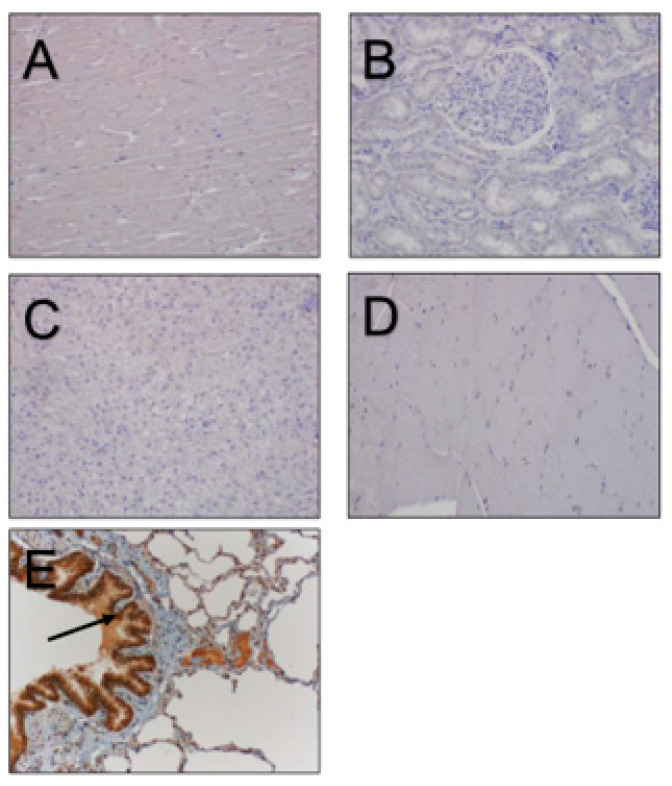
Non-reproductive tract tissues stained for β-NGF: heart (**A**), kidney (**B**), liver (**C**), skeletal muscle (**D**), and lung (**E**). Note the heavy staining in the bronchial epithelial cells of the lung ((**E**), arrow) and an absence of staining in the other tissue.

No differences in immunostaining of β-NGF were noticed between the one-day-old foal, the one-year-old colt, or the adult reproductive tracts. Immunostaining for β-NGF in the non-reproductive tract tissues, including the heart, lung, spleen, skeletal muscle, and kidney, were all negative, except for lung tissue. Positive staining was observed in the bronchial epithelial cells (Figure 5). The β-NGF protein block, evaluated on all of the reproductive tissues, resulted in the complete blockage of immunostaining in all tissues, with the exception of only a limited blockage in testicular tissue.

## 4. Discussion

The testes of stallions exhibited the strongest immunostaining for β-NGF in the efferent ducts, with light, scattered immunostaining noted in the interstitial (Leydig) cells, Sertoli cells, spermatogonia, and smooth muscle. In the bull testis, immunoreactivity was localized in the myoid epithelial cells surrounding the seminiferous tubules, whereas spermatids and interstitial cells were negative for β-NGF staining [13]. In camelids and cervids, low intensity staining was noted in the interstitial cells, smooth muscle, and connective tissue within the testis [14].

In the present study, no differences in immunostaining for β-NGF was noted between testicular tissue of male horses of different ages. In a study of age-associated β-NGF immunostaining in alpacas, Wang and colleagues noted positive immunostaining in the Sertoli cells, spermatogonia, and primary spermatocytes of a 1-month-old cria, whereas a 12-month-old male alpaca showed strongest staining in the spermatogenic cells, Sertoli cells, and in scattered interstitial cells [15]. Testes from a 24-month-old alpaca stained positive in the stromal cells, Sertoli cells, and germ cells, but the intensity was less than that of the 12-month-old alpaca. It was suggested that β-NGF could play a role in pubertal development of the testes and spermatogonia of the male alpaca. Sanchez-Rodriguez and co-workers compared the immunolocalization of β-NGF and its receptor TrkA in the accessory sex glands and epididymis of 22- and 37-week-old rabbits [16]. It was noted that β-NGF was abundant in the prostate at both developmental stages, but the staining intensity differed in location with the epithelial cells at the two ages. In the present study, reproductive tissues were only available from a very limited number of young male horses. Immunostaining for β-NGF in the 2 young horses was evident in all reproductive tissue evaluated, and the intensity was similar to that of adult stallions. Due to the limited sample size, we are unable to draw any conclusions on the role of β-NGF in development of the reproductive tract, spermatogenesis, or epididymal function in male horses.

Epididymal tissue of the stallion exhibited strong cytoplasmic and nuclear immunostaining in the apical region of the epithelium throughout the head, body, and tail. The intensity of the immunostaining increased distally from the head to the tail of the epididymis, with the strongest staining located in the epididymal tail, which continued into the vas deferens. Bulls have also been reported to exhibit immunostaining for β-NGF in the epithelium of the epididymis [13]. Camelids, cervids, and rats were noted to exhibit faint to strong immunostaining in the connective tissue of the epididymis [13].

In the stallion, all four accessory sex glands exhibited immunostaining against β-NGF. Bulls were noted to exhibit immunostaining for β-NGF in the ampullae, seminal vesicles, and prostate, but not in the bulbourethral gland [10,13]. In the rabbit, strong immunostaining for β-NGF was present in the prostate, with limited staining in the bulbourethral glands [16]. Strong epithelial staining throughout the accessory sex glands of the stallion suggests that β-NGF is secreted into the seminal plasma. As noted previously, β-NGF produced with the reproductive tract of male camelids is secreted into the seminal plasma and has a physiologic role in inducing ovulation in the female [17,18]. In the cow, a species with spontaneous ovulations, β-NGF derived from bull seminal plasma was reported to improve synchronization of ovulation and enhanced luteal development [19].

Mares are also spontaneous ovulators, and a very high percentage of mares ovulate late in estrus during the physiologic breeding season, whether or not they were mated by a stallion. Administration of human chorionic gonadotropin (hCG) and agonists of gonadotropin releasing hormone (GnRH) are commonly used to induce ovulation for reproductive management purposes. Apparently, injection of stallion seminal plasma or β-NGF purified from stallion seminal plasma into estrual mares has not been reported. However, systemic administration of stallion seminal plasma has been used with limited success to stimulate ovulation in llamas [14]. Clearly additional research is needed to determine if β-NGF present in stallion seminal plasma plays any physiologic role in the mare in either the modulation of gonadotropin secretion or in the induction of ovulation.

## 5. Conclusions

In conclusion, β-NGF was detected in multiple sites within the stallion reproductive tract. The primary locations of β-NGF immunoreactivity were the efferent ducts of the testes and the apical epithelium of all four accessory sex glands.

## Figures and Tables

**Table 1:** β-NGF staining intensity in the four adult stallion reproductive tracts. Relative staining intensities were graded + (weak to moderate), ++ (strong), +++ (very strong).

Reproductive Tissue	NGF-β Staining Intensity
Sertoli Cells of Testes	+
Leydig Cells of Testes (interstitial)	+
Efferent Duct of Testes	+++
Head of Epididymis	+
Body of Epididymis	+
Tail of Epididymis	++
Vas Deferens	++
Ampulla	+++
Seminal Vesicle	+++
Prostate Gland	+++
Bulbourethral Gland	+++

## Data Availability

The raw data supporting the conclusions of this article will be made available by the corresponding author on request.

## References

[1] Mann T. (1964). Male accessory organs of reproduction, and their secretory product: The seminal plasma. The Biochemistry of Semen and of the Male Reproductive Tract.

[2] Amann R.P., McKinnon A.O., Squires E.L., Vaala W.E., Varner D.D. (2011). Functional anatomy of the adult male. Equine Reproduction.

[3] Adams G., Ratto M., Silva M., Carrasco R. (2016). Ovulation-inducing factor (OIF/NGF) in seminal plasma: A review and update. Reprod. Domest. Anim..

[4] Druart X., Rickard J.P., Mactier S., Kohnke P.L., Kershaw-Young C.M., Bathgate R., Gibb Z., Crossett B., Tsikis G., Labas V. (2013). Proteomic characterization and cross species comparison of mammalian seminal plasma. J. Proteom..

[5] Chen B., Yuen Z., Pan G. (1985). Semen-induced ovulation in the bactrian camel (*Camelus bactrianus*). Reproduction.

[6] Xu Y., Wang H., Zeng G., Jiang G., Gao Y. (1985). Hormone concentrations before and after semen-induced ovulation in the Bactrian camel (*Camelus bactrianus*). J. Reprod. Fertil..

[7] Pan G., Zhao X., Chen S., Jiang S., Huang Y., Zu Y., Wang H. The ovulation inducing effect of seminal plasma in the Bactrian camel. Proceedings of the First International Camel Conference.

[8] Adams G.P., Ratto M.H., Huanca W., Singh J. (2005). Ovulation-inducing factor in the seminal plasma of alpacas and llamas. Biol. Reprod..

[9] Ratto M.H., Leduc Y.A., Valderrama X.P., Van Straaten K.E., Delbaere L.T.J., Pierson R.A., Adams G.P. (2012). The nerve of ovulation-inducing factor in semen. Proc. Nat. Acad. Sci. USA.

[10] Harper G.P., Thoenen H. (1980). The distribution of nerve growth factor in the male sex organs of mammals. J. Neurochem..

[11] Thoenen H., Barde Y.A. (1980). Physiology of nerve growth factor. Physiol. Rev..

[12] Shikata H., Utsumi N., Hiramatsu M., Minami N., Nemoto N., Shikata T. (1984). Immunohistochemical localization of nerve growth factor and epidermal growth factor in guinea pig prostate gland. Histochemistry.

[13] Bogle O.A., Carrasco R.A., Ratto M.H., Singh J., Adams G.P. (2018). Source and localization of ovulation-inducing factor/nerve growth factor in male reproductive tissues among mammalian species. Biol. Reprod..

[14] Bogle O.A., Ambati D., Davis R.P., Adams G.P. (2009). Evidence for the presence of ovulation-inducing factor in porcine and equine seminal plasma. Reprod. Fertil. Dev..

[15] Wang H., Dong Y., Chen W., Hei J., Dong C. (2011). Expression and localization of nerve growth factor (NGF) in the testis of alpaca (*Llama pacos*). Folia. Histochem. Cytobiol..

[16] Sanchez-Rodriguez A., Arias-Alvarez M., Timón P., Bautista J.M., Rebollar P.G., Lorenzo P.L., Garcia-Garcia R.M. (2019). Characterization of β-Nerve Growth Factor-TrkA system in male reproductive tract of rabbit and the relationship between β-NGF and testosterone levels with seminal quality during sexual maturation. Theriogenology.

[17] Pan G., Chen Z., Liu X., Li D., Xie Q., Ling F., Fang L. (2001). Isolation and purification of the ovulation-inducing factor from seminal plasma in the bactrian camel (*Camelus bactrianus*). Theriogenology.

[18] Meriem F., José Alvaro C.P., Imed S., Rosaura P.P., Mouldi S.M., Adriana C., Touhami K., Teresa M.B., Mohamed H. (2017). Identification of β-nerve growth factor in dromedary camel seminal plasma and its role in induction of ovulation in females. Emir. J. Food Agric..

[19] Tribulo P., Bogle O., Mapletoft R.J., Adams G.P. (2015). Bioactivity of ovulation inducing factor (or nerve growth factor) in bovine seminal plasma and its effects on ovarian function in cattle. Theriogenology.

